# Aqua­(2,2′-bipyridine-κ^2^ *N*,*N*′)bis­(thio­phene-2-carboxyl­ato-κ*O*)copper(II)

**DOI:** 10.1107/S1600536809026713

**Published:** 2009-07-11

**Authors:** Reda F. Mahmoud, Christoph Janiak

**Affiliations:** a Institut für Anorganische und Analytische Chemie, Universität Freiburg, Albertstrasse 21, D-79104 Freiburg, Germany

## Abstract

In the title complex, [Cu(C_5_H_3_O_2_S)_2_(C_10_H_8_N_2_)(H_2_O)], the Cu^II^ atom is in a distorted square-pyramidal environment, with an Addison τ parameter of 0.07. The coordination geometry is defined by two nitro­gen donors from the 2,2′-bipyridine ligand, two O atoms from two monodentate thio­phene-2-carboxyl­ate ligands and one O atom from the aqua ligand. The latter occupies the elongated apical position. This is different from the related structure of aqua­(1,10-phenanthroline)bis­(thio­phene-2-carboxyl­ato)copper(II) where a carboxyl­ate O atom is in the apical position [Feng *et al.* (2005 ▶). *Z. Kristallogr. New Cryst. Struct.* **220**, 429–430]. The uncoordinated carboxyl­ate O atoms form intra- and inter­molecular hydrogen bonds to the aqua ligand. Two neighbouring 2,2′-bipyridine ligands form a π-stack, with a centroid–centroid distance of 3.683 (2) Å.

## Related literature

Thio­phenes substituted in the 2-position are an important constituent of the drugs methapyrilene, temidap, tienilic acid and temocillin (Rance & Damani, 1989 ▶). Metal complexes containing the thio­phene unit have exhibited enhanced anti-amoebic activity (Bharti *et al.*, 2003 ▶). For the use of thio­phene-2-carboxylic acid (Htpc) to prepare single mol­ecular magnet (SMM) and photoluminescence materials, see: Kuroda-Sowa *et al.* (2003 ▶); Teotonio *et al.* (2004 ▶). For the thermal behavior of metal–tpc complexes, see: Lumme & Korvola (1975 ▶). For the structures of 2-thio­phene­carboxyl­ate complexes, see: Feng *et al.* (2005 ▶); Panagoulis *et al.* (2007 ▶); Byrnes *et al.* (2004 ▶); Yin & Sun (2005 ▶); Yin *et al.* (2004 ▶). For hydrogen bonds from the aqua ligand to uncoordinated carboxyl O atoms, see: Habib & Janiak (2008 ▶); Wisser & Janiak (2007*a* ▶,*b* ▶); Janiak (2000 ▶). For details of the Addison τ parameter, see: Addison *et al.* (1984 ▶).
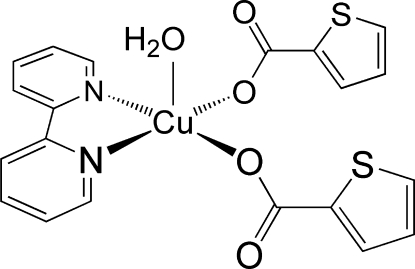


## Experimental

### 

#### Crystal data

[Cu(C_5_H_3_O_2_S)_2_(C_10_H_8_N_2_)(H_2_O)]
*M* *_r_* = 492.01
Monoclinic, 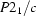

*a* = 6.8458 (5) Å
*b* = 18.3799 (15) Å
*c* = 16.8421 (12) Å
β = 101.5164 (19)°
*V* = 2076.5 (3) Å^3^
*Z* = 4
Mo *K*α radiation
μ = 1.29 mm^−1^
*T* = 123 K
0.35 × 0.22 × 0.18 mm

#### Data collection

Rigaku R-AXIS Spider image-plate detector diffractometer
Absorption correction: multi-scan (**ABSCOR**; Higashi, 1995 ▶) *T*_min_ = 0.661, *T*_max_ = 0.801
32855 measured reflections
4224 independent reflections
3637 reflections with *I* > 2σ(*I*)
*R*_int_ = 0.034

#### Refinement

*R*[*F* ^2^ > 2σ(*F* ^2^)] = 0.037
*wR*(*F* ^2^) = 0.099
*S* = 1.05
4224 reflections
277 parameters
H atoms treated by a mixture of independent and constrained refinement
Δρ_max_ = 0.64 e Å^−3^
Δρ_min_ = −0.60 e Å^−3^

### 

Data collection: *CrystalClear* (Rigaku, 2007 ▶); cell refinement: *CrystalClear*; data reduction: *CrystalClear*; program(s) used to solve structure: *SHELXS97* (Sheldrick, 2008 ▶); program(s) used to refine structure: *SHELXL97* (Sheldrick, 2008 ▶); molecular graphics: *DIAMOND* (Crystal Impact, 2009 ▶); software used to prepare material for publication: *publCIF* (Westrip, 2009 ▶).

## Supplementary Material

Crystal structure: contains datablocks I, global. DOI: 10.1107/S1600536809026713/fj2230sup1.cif

Structure factors: contains datablocks I. DOI: 10.1107/S1600536809026713/fj2230Isup2.hkl

Additional supplementary materials:  crystallographic information; 3D view; checkCIF report

## Figures and Tables

**Table:** 

Cu—O1	1.9447 (18)
Cu—O3	1.9909 (19)
Cu—N1	2.011 (2)
Cu—N2	2.018 (2)
Cu—O5	2.236 (2)

**Table:** 

O1—Cu—O3	90.18 (8)
O1—Cu—N1	167.13 (8)
O3—Cu—N1	94.08 (8)
O1—Cu—N2	92.15 (8)
O3—Cu—N2	163.16 (8)
N1—Cu—N2	80.33 (8)
O1—Cu—O5	92.18 (8)
O3—Cu—O5	99.70 (8)
N1—Cu—O5	99.04 (8)
N2—Cu—O5	96.87 (8)

**Table 2:** Hydrogen-bond geometry (Å, °)

*D*—H⋯*A*	*D*—H	H⋯*A*	*D*⋯*A*	*D*—H⋯*A*
O5—H5*A*⋯O2	0.73 (4)	1.99 (4)	2.682 (3)	160 (4)
O5—H5*B*⋯O4^i^	0.73 (4)	2.02 (4)	2.741 (3)	171 (4)

Symmetry code: (i) 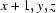.

**Fig. 1.**
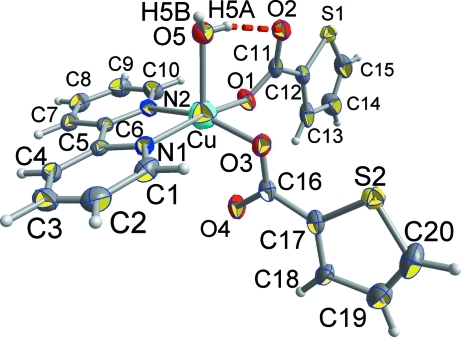
: Asymmetric molecular unit of [Cu(C4H3SCOO)2(C10H8N2)(H2O)] in a perspective view with thermal ellipsoids (at 50% probability); intramolecular hydrogen bond as dashed line.

**Fig. 2.**
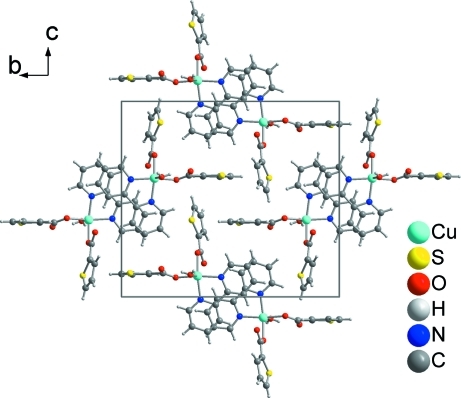
: Crystal packing of [Cu(C4H3SCOO)2(C10H8N2)(H2O)] projected onto the bc-plane.

## supplementary crystallographic information

### Comment

Thiophenes substituted in the 2-position are an important constituent of the drugs methapyrilene, temidap, tienilic acid and temocillin (Rance & Damani, 1989). Metal complexes containing the thiophene moiety have exhibited enhanced antiamoebic activity (Bharti *et al.*, 2003). Knowledge of the structural peculiarities of a biologically active molecule and its inherent 3 -dimensional structure is a necessary condition for investigating the interaction with metal ions and for designing new compounds. Recently, thiophene-2-carboxylic acid (Htpc) has been used to prepare single molecular magnet (SMM) and photoluminescence materials (Kuroda-Sowa *et al.*, 2003; Teotonio *et al.*, 2004). The thermal behavior of metal-tpc complexes was studied (Lumme & Korvola, 1975). Single crystal structures of 2- thiophenecarboxylate complexes are still limited (Feng *et al.*, 2005), with recent additions of a tpc-bridged di-copper (Panagoulis *et al.*, 2007), di-molybdenum (Byrnes *et al.*, 2004), di-terbium and di-europium complex (Yin & Sun, 2005; Yin *et al.*, 2004).

The molecular structure of the title complex is shown in Fig. 1. The Cu atoms are in a square-pyramidal environment with a long apical Cu—OH_2_ bond due to the Jahn-Teller effect. No relevant π—π or C—H···π interactions are found between the thiophene rings only between bipyridine ligands (Fig. 2). There, the π-stacking interactions can be viewed as strong because of the rather short centroid-centroid contacts (3.683 Å), small slip angles (22.9°) and short interplanar separation (3.4 Å) which translate into a sizable overlap of the near parallel aromatic planes (interplanar angle 2.5°) (Janiak, 2000). The intra- and intermolecular hydrogen bonds from the aqua ligand to the uncoordinated carboxyl oxygen atoms are normal (Habib & Janiak, 2008; Wisser & Janiak, 2007*a*; Wisser & Janiak, 2007*b*).

### Experimental

A mixture of copper acetate, Cu(CH_3_COO)_2_. H_2_O (57.9 mg, 0.29 mmol) and thiophene-2-carboxylic acid, Htpc (76.9 mg, 0.6 mmol) in 10 ml water was added to a 10 ml CH_3_OH solution of 2,2'-bipyridine (48.4 mg, 0.31 mmol). Then the resulting solution was set aside and the solvent allowed to evaporate at room temperature. After three days, blue rod-shaped crystals were obtained in (yield 99 mg, 32% based on Htpc). Elemental analysis C_22_H_16_CuN_2_O_5_S_2_(516.05) calcd. C 51.20, H 3.13, N 5.43, S 12.43; found: C 50.97, H 3.16, N 5.33, S 12.20%.

### Refinement

Hydrogen atoms for aromatic CH were positioned geometrically (C—H = 0.94 Å) and refined using a riding model. Protic hydrogen atoms of the aqua ligand were found and refined with *U*_iso_(H) = 1.5*U*_eq_(O).

### Crystal data

**Table:**

[Cu(C_5_H_3_O_2_S)_2_(C_10_H_8_N_2_)(H_2_O)]	*F*(000) = 1004
*M**_r_* = 492.01	*D*_x_ = 1.574 Mg m^−^^3^
Monoclinic, *P*2_1_/*c*	Mo *K*α radiation, λ = 0.71073 Å
Hall symbol: -P 2ybc	Cell parameters from 28386 reflections
*a* = 6.8458 (5) Å	θ = 3.0–27.5°
*b* = 18.3799 (15) Å	µ = 1.29 mm^−^^1^
*c* = 16.8421 (12) Å	*T* = 123 K
β = 101.5164 (19)°	Column, blue
*V* = 2076.5 (3) Å^3^	0.35 × 0.22 × 0.18 mm
*Z* = 4	

### Data collection

**Table:**

Rigaku R-AXIS Spider image-plate detector diffractometer	4224 independent reflections
Radiation source: fine-focus sealed tube	3637 reflections with *I* > 2σ(*I*)
graphite	*R*_int_ = 0.034
ω scans	θ_max_ = 26.4°, θ_min_ = 3.0°
Absorption correction: multi-scan (*ABSCOR*; Higashi, 1995)	*h* = −8→8
*T*_min_ = 0.661, *T*_max_ = 0.801	*k* = −22→22
32855 measured reflections	*l* = −21→21

### Refinement

**Table:**

Refinement on *F*^2^	Primary atom site location: structure-invariant direct methods
Least-squares matrix: full	Secondary atom site location: difference Fourier map
*R*[*F*^2^ > 2σ(*F*^2^)] = 0.037	Hydrogen site location: inferred from neighbouring sites
*wR*(*F*^2^) = 0.099	H atoms treated by a mixture of independent and constrained refinement
*S* = 1.05	*w* = 1/[σ^2^(*F*_o_^2^) + (0.0489*P*)^2^ + 2.2127*P*] where *P* = (*F*_o_^2^ + 2*F*_c_^2^)/3
4224 reflections	(Δ/σ)_max_ < 0.001
277 parameters	Δρ_max_ = 0.64 e Å^−^^3^
0 restraints	Δρ_min_ = −0.59 e Å^−^^3^

### Special details

**Table:**

Experimental. IR (ATR): 3315 (m, br, νO-H, H-bonded), 3075 (m, sh, νC-H, aromatic), 3115 (m, sh) 1557 (s, sh, ν*_asym_*CO_2_, ionically bonded to COO-Cu), 1520 (s, sh, ν*_asym_*CO_2_, intramolecularly H-bonded), 1470 (m, sh) 1422 (s, sh) 1370 (s, sh, ν*_sym_*CO_2_), 1336 (m, sh) (νC-O, free), 1312 (w, br, νC-O^···^H—O, H-bonded), 1224 (m, sh, νC-O), 1115 (s, sh, νC-N), 1056 (m, sh), 1026 (s, sh), 982 (m, sh), 911 (m, sh), 860 (s, sh), 808 (m, sh), 770 (s, sh), 713 (s, sh), 659 (w, sh), 631 (w, br), 539 (w, sh, νCu-O), 506 (m, sh), 461 (m, sh), 412 (s, sh, νCu-N) cm^-1^.
Geometry. All e.s.d.'s (except the e.s.d. in the dihedral angle between two l.s. planes) are estimated using the full covariance matrix. The cell e.s.d.'s are taken into account individually in the estimation of e.s.d.'s in distances, angles and torsion angles; correlations between e.s.d.'s in cell parameters are only used when they are defined by crystal symmetry. An approximate (isotropic) treatment of cell e.s.d.'s is used for estimating e.s.d.'s involving l.s. planes.
Refinement. Refinement of *F*^2^ against ALL reflections. The weighted *R*-factor *wR* and goodness of fit *S* are based on *F*^2^, conventional *R*-factors *R* are based on *F*, with *F* set to zero for negative *F*^2^. The threshold expression of *F*^2^ > σ(*F*^2^) is used only for calculating *R*-factors(gt) *etc*. and is not relevant to the choice of reflections for refinement. *R*-factors based on *F*^2^ are statistically about twice as large as those based on *F*, and *R*- factors based on ALL data will be even larger.

### Fractional atomic coordinates and isotropic or equivalent isotropic displacement parameters (Å^2^)

**Table:**

	*x*	*y*	*z*	*U*_iso_*/*U*_eq_	
Cu	0.67488 (4)	0.151034 (16)	0.393556 (18)	0.02638 (11)	
S1	0.63575 (13)	0.46085 (4)	0.37885 (5)	0.0465 (2)	
S2	0.31914 (12)	0.17767 (6)	0.10820 (5)	0.0497 (2)	
O1	0.5925 (3)	0.25157 (10)	0.40169 (12)	0.0373 (4)	
O2	0.8271 (3)	0.31699 (10)	0.35780 (12)	0.0355 (4)	
O3	0.5534 (3)	0.15076 (10)	0.27585 (11)	0.0331 (4)	
O4	0.2930 (3)	0.12147 (11)	0.32959 (11)	0.0351 (4)	
O5	0.9860 (3)	0.18398 (12)	0.38761 (14)	0.0391 (5)	
H5A	0.964 (6)	0.221 (2)	0.374 (2)	0.059*	
H5B	1.062 (6)	0.168 (2)	0.368 (2)	0.059*	
N1	0.7125 (3)	0.04250 (12)	0.39954 (12)	0.0266 (4)	
N2	0.7215 (3)	0.13725 (11)	0.51474 (12)	0.0277 (4)	
C1	0.7038 (4)	−0.00185 (15)	0.33635 (17)	0.0340 (6)	
H1	0.6855	0.0186	0.2836	0.041*	
C2	0.7204 (4)	−0.07660 (16)	0.34483 (19)	0.0391 (6)	
H2	0.7140	−0.1069	0.2987	0.047*	
C3	0.7463 (4)	−0.10622 (15)	0.4214 (2)	0.0395 (7)	
H3	0.7583	−0.1574	0.4288	0.047*	
C4	0.7545 (4)	−0.06063 (14)	0.48737 (18)	0.0324 (6)	
H4	0.7713	−0.0802	0.5405	0.039*	
C5	0.7381 (3)	0.01361 (13)	0.47514 (15)	0.0256 (5)	
C6	0.7470 (3)	0.06754 (13)	0.54036 (15)	0.0252 (5)	
C7	0.7786 (4)	0.05039 (16)	0.62249 (16)	0.0331 (6)	
H7	0.7931	0.0011	0.6398	0.040*	
C8	0.7884 (4)	0.10584 (18)	0.67850 (17)	0.0398 (7)	
H8	0.8117	0.0951	0.7348	0.048*	
C9	0.7643 (5)	0.17670 (18)	0.65216 (18)	0.0423 (7)	
H9	0.7712	0.2155	0.6899	0.051*	
C10	0.7296 (4)	0.19039 (15)	0.56982 (17)	0.0371 (6)	
H10	0.7108	0.2393	0.5516	0.045*	
C11	0.6649 (4)	0.30992 (14)	0.37863 (14)	0.0289 (5)	
C12	0.5375 (4)	0.37527 (14)	0.38065 (15)	0.0300 (5)	
C13	0.3272 (5)	0.37346 (17)	0.38295 (17)	0.0406 (7)	
H13	0.2457	0.3316	0.3830	0.049*	
C14	0.2658 (5)	0.45070 (18)	0.3853 (2)	0.0508 (8)	
H14	0.1336	0.4647	0.3878	0.061*	
C15	0.4127 (5)	0.49975 (17)	0.3835 (2)	0.0488 (8)	
H15	0.3930	0.5509	0.3847	0.059*	
C16	0.3672 (4)	0.13855 (13)	0.27060 (15)	0.0285 (5)	
C17	0.2351 (4)	0.14483 (13)	0.18962 (16)	0.0300 (5)	
C18	0.0315 (4)	0.12344 (15)	0.16881 (17)	0.0351 (6)	
H18	−0.0438	0.1036	0.2053	0.042*	
C19	−0.0425 (5)	0.13653 (18)	0.0838 (2)	0.0492 (8)	
H19	−0.1754	0.1259	0.0573	0.059*	
C20	0.0926 (5)	0.1648 (2)	0.04594 (19)	0.0513 (8)	
H20	0.0657	0.1767	−0.0101	0.062*	

### Atomic displacement parameters (Å^2^)

**Table:**

	*U*^11^	*U*^22^	*U*^33^	*U*^12^	*U*^13^	*U*^23^
Cu	0.02852 (17)	0.02612 (17)	0.02524 (17)	0.00260 (12)	0.00714 (12)	0.00575 (12)
S1	0.0513 (5)	0.0344 (4)	0.0543 (5)	0.0013 (3)	0.0115 (4)	0.0014 (3)
S2	0.0458 (4)	0.0696 (6)	0.0346 (4)	0.0073 (4)	0.0100 (3)	0.0159 (4)
O1	0.0436 (11)	0.0269 (9)	0.0451 (11)	0.0056 (8)	0.0177 (9)	0.0092 (8)
O2	0.0343 (10)	0.0339 (10)	0.0397 (11)	0.0042 (8)	0.0108 (8)	0.0075 (8)
O3	0.0298 (9)	0.0420 (11)	0.0278 (9)	0.0031 (8)	0.0064 (7)	0.0086 (8)
O4	0.0352 (10)	0.0409 (11)	0.0318 (10)	0.0069 (8)	0.0131 (8)	0.0117 (8)
O5	0.0311 (10)	0.0376 (11)	0.0523 (13)	0.0088 (9)	0.0172 (9)	0.0111 (10)
N1	0.0231 (10)	0.0294 (11)	0.0269 (11)	0.0020 (8)	0.0041 (8)	0.0024 (8)
N2	0.0280 (10)	0.0299 (11)	0.0261 (11)	−0.0029 (9)	0.0078 (8)	0.0020 (9)
C1	0.0312 (13)	0.0391 (14)	0.0308 (14)	0.0027 (11)	0.0042 (11)	−0.0032 (11)
C2	0.0318 (14)	0.0390 (15)	0.0462 (17)	0.0003 (12)	0.0070 (12)	−0.0111 (13)
C3	0.0303 (14)	0.0276 (13)	0.0612 (19)	−0.0002 (11)	0.0102 (13)	−0.0010 (13)
C4	0.0243 (12)	0.0282 (13)	0.0446 (15)	0.0018 (10)	0.0061 (11)	0.0085 (11)
C5	0.0163 (10)	0.0302 (12)	0.0298 (12)	−0.0006 (9)	0.0031 (9)	0.0050 (10)
C6	0.0186 (11)	0.0306 (12)	0.0265 (12)	−0.0020 (9)	0.0047 (9)	0.0050 (10)
C7	0.0273 (12)	0.0415 (15)	0.0300 (13)	−0.0001 (11)	0.0044 (10)	0.0094 (11)
C8	0.0358 (15)	0.0585 (18)	0.0254 (13)	−0.0037 (13)	0.0064 (11)	0.0026 (13)
C9	0.0448 (16)	0.0499 (17)	0.0327 (15)	−0.0066 (14)	0.0088 (12)	−0.0107 (13)
C10	0.0449 (16)	0.0333 (14)	0.0347 (15)	−0.0039 (12)	0.0115 (12)	−0.0035 (12)
C11	0.0346 (14)	0.0301 (13)	0.0207 (12)	0.0040 (11)	0.0024 (10)	0.0043 (10)
C12	0.0379 (14)	0.0268 (12)	0.0257 (12)	0.0017 (11)	0.0073 (10)	0.0048 (10)
C13	0.0475 (16)	0.0420 (16)	0.0388 (15)	0.0290 (14)	0.0243 (13)	0.0167 (13)
C14	0.0514 (19)	0.0436 (17)	0.064 (2)	0.0156 (15)	0.0272 (17)	0.0092 (15)
C15	0.063 (2)	0.0322 (15)	0.0548 (19)	0.0135 (14)	0.0199 (16)	0.0066 (14)
C16	0.0322 (13)	0.0258 (12)	0.0285 (13)	0.0059 (10)	0.0088 (10)	0.0048 (10)
C17	0.0350 (14)	0.0272 (12)	0.0280 (13)	0.0054 (11)	0.0067 (11)	0.0058 (10)
C18	0.0321 (13)	0.0338 (14)	0.0334 (14)	0.0017 (11)	−0.0081 (11)	0.0038 (11)
C19	0.0461 (17)	0.0504 (18)	0.0441 (18)	−0.0021 (15)	−0.0081 (14)	0.0014 (15)
C20	0.056 (2)	0.066 (2)	0.0277 (15)	0.0155 (17)	−0.0012 (14)	0.0041 (14)

### Geometric parameters (Å, °)

**Table:**

Cu—O1	1.9447 (18)	C4—C5	1.381 (3)
Cu—O3	1.9909 (19)	C4—H4	0.9500
Cu—N1	2.011 (2)	C5—C6	1.472 (4)
Cu—N2	2.018 (2)	C6—C7	1.393 (3)
Cu—O5	2.236 (2)	C7—C8	1.381 (4)
S1—C15	1.702 (3)	C7—H7	0.9500
S1—C12	1.714 (3)	C8—C9	1.375 (4)
S2—C17	1.700 (3)	C8—H8	0.9500
S2—C20	1.706 (3)	C9—C10	1.383 (4)
O1—C11	1.274 (3)	C9—H9	0.9500
O2—C11	1.236 (3)	C10—H10	0.9500
O3—C16	1.280 (3)	C11—C12	1.489 (4)
O4—C16	1.243 (3)	C12—C13	1.448 (4)
O5—H5A	0.73 (4)	C13—C14	1.483 (4)
O5—H5B	0.73 (4)	C13—H13	0.9500
N1—C1	1.332 (3)	C14—C15	1.355 (5)
N1—C5	1.358 (3)	C14—H14	0.9500
N2—C10	1.340 (3)	C15—H15	0.9500
N2—C6	1.352 (3)	C16—C17	1.483 (4)
C1—C2	1.384 (4)	C17—C18	1.423 (4)
C1—H1	0.9500	C18—C19	1.440 (4)
C2—C3	1.378 (4)	C18—H18	0.9500
C2—H2	0.9500	C19—C20	1.330 (5)
C3—C4	1.384 (4)	C19—H19	0.9500
C3—H3	0.9500	C20—H20	0.9500
			
O1—Cu—O3	90.18 (8)	C8—C7—H7	120.4
O1—Cu—N1	167.13 (8)	C6—C7—H7	120.4
O3—Cu—N1	94.08 (8)	C9—C8—C7	119.5 (3)
O1—Cu—N2	92.15 (8)	C9—C8—H8	120.3
O3—Cu—N2	163.16 (8)	C7—C8—H8	120.3
N1—Cu—N2	80.33 (8)	C8—C9—C10	118.8 (3)
O1—Cu—O5	92.18 (8)	C8—C9—H9	120.6
O3—Cu—O5	99.70 (8)	C10—C9—H9	120.6
N1—Cu—O5	99.04 (8)	N2—C10—C9	122.5 (3)
N2—Cu—O5	96.87 (8)	N2—C10—H10	118.8
C15—S1—C12	91.48 (15)	C9—C10—H10	118.8
C17—S2—C20	91.96 (15)	O2—C11—O1	126.9 (2)
C11—O1—Cu	129.96 (17)	O2—C11—C12	119.0 (2)
C16—O3—Cu	106.35 (16)	O1—C11—C12	114.0 (2)
Cu—O5—H5A	97 (3)	C13—C12—C11	124.9 (2)
Cu—O5—H5B	133 (3)	C13—C12—S1	114.7 (2)
H5A—O5—H5B	110 (4)	C11—C12—S1	120.4 (2)
C1—N1—C5	119.1 (2)	C12—C13—C14	105.5 (3)
C1—N1—Cu	125.59 (18)	C12—C13—H13	127.3
C5—N1—Cu	115.21 (17)	C14—C13—H13	127.3
C10—N2—C6	119.0 (2)	C15—C14—C13	114.9 (3)
C10—N2—Cu	125.80 (18)	C15—C14—H14	122.5
C6—N2—Cu	115.21 (17)	C13—C14—H14	122.5
N1—C1—C2	122.4 (3)	C14—C15—S1	113.4 (2)
N1—C1—H1	118.8	C14—C15—H15	123.3
C2—C1—H1	118.8	S1—C15—H15	123.3
C3—C2—C1	118.8 (3)	O4—C16—O3	123.3 (2)
C3—C2—H2	120.6	O4—C16—C17	118.9 (2)
C1—C2—H2	120.6	O3—C16—C17	117.8 (2)
C2—C3—C4	119.3 (3)	C18—C17—C16	126.3 (2)
C2—C3—H3	120.4	C18—C17—S2	111.8 (2)
C4—C3—H3	120.4	C16—C17—S2	121.8 (2)
C5—C4—C3	119.3 (3)	C17—C18—C19	109.4 (3)
C5—C4—H4	120.4	C17—C18—H18	125.3
C3—C4—H4	120.4	C19—C18—H18	125.3
N1—C5—C4	121.2 (2)	C20—C19—C18	113.7 (3)
N1—C5—C6	114.5 (2)	C20—C19—H19	123.2
C4—C5—C6	124.3 (2)	C18—C19—H19	123.2
N2—C6—C7	121.1 (2)	C19—C20—S2	113.2 (2)
N2—C6—C5	114.6 (2)	C19—C20—H20	123.4
C7—C6—C5	124.3 (2)	S2—C20—H20	123.4
C8—C7—C6	119.2 (3)		

### Hydrogen-bond geometry (Å, °)

**Table:**

*D*—H···*A*	*D*—H	H···*A*	*D*···*A*	*D*—H···*A*
O5—H5A···O2	0.73 (4)	1.99 (4)	2.682 (3)	160 (4)
O5—H5B···O4^i^	0.73 (4)	2.02 (4)	2.741 (3)	171 (4)

Symmetry codes: (i) *x*+1, *y*, *z*.
